# Development of an optimized risk score to predict short‐term death among acute myocardial infarction patients in rural China

**DOI:** 10.1002/clc.23598

**Published:** 2021-03-25

**Authors:** Sheng‐ji Wang, Zhen‐Xiu Cheng, Xiao‐ting Fan, Yong‐gang Lian

**Affiliations:** ^1^ Emergency Department Linyi People's Hospital Affiliated to Shandong University Linyi Shandong China; ^2^ Department of Neurosurgery Linyi People's Hospital Affiliated to Shandong University Linyi Shandong China; ^3^ Department of Neurosurgery ICU Linyi People's Hospital Affiliated to Shandong University Linyi Shandong China

**Keywords:** acute myocardial infarction, risk prediction, short‐term mortality, score system

## Abstract

**Background:**

Risk stratification of patients with acute myocardial infarction (AMI) is of great clinical significance.

**Hypothesis:**

The present study aimed to establish an optimized risk score to predict short‐term (6‐month) death among rural AMI patients from China.

**Methods:**

We enrolled 6581 AMI patients and extracted relevant data. Patients were divided chronologically into a derivation cohort (*n* = 5539), to establish the multivariable risk prediction model, and a validation cohort (*n* = 1042), to validate the risk score.

**Results:**

Six variables were identified as independent predictors of short‐term death and were used to establish the risk score: age, Killip class, blood glucose, creatinine, pulmonary artery systolic pressure, and percutaneous coronary intervention treatment. The area under the ROC curve (AUC) of the optimized risk score was 0.82 within the derivation cohort and 0.81 within the validation cohort. The diagnostic performance of the optimized risk score was superior to that of the GRACE risk score (AUC 0.76 and 0.75 in the derivation and validation cohorts, respectively; p < .05).

**Conclusion:**

These results indicate that the optimized scoring method developed here is a simple and valuable instrument to accurately predict the risk of short‐term mortality in rural patients with AMI.

## 1 INTRODUCTION

Coronary artery disease (CAD) has become the leading contributor to disease burden worldwide.^1^ Acute myocardial infarction (AMI), the most severe manifestation of CAD, causes more than 2.4 million deaths in the USA and over 4 million deaths in Europe and China annually.^2^ In China, AMI continues to be the leading cause of death, overcoming cancer and other diseases; meanwhile, the incidence and mortality of AMI in rural areas have exceeded those in cities.^3^ However, there are very few epidemiological studies focusing on AMI in patients from rural areas in China.

AMI involves a wide spectrum of clinical presentations and prognosis.^4^ Risk stratification for patients with AMI is of clinical significance, and early accurate prognosis is useful for selecting an appropriate level of care and optimal pharmacological or invasive treatment. Guidelines from both the American College of Cardiology/American Heart Association (ACC/AHA)^4^ and the European Society of Cardiology (ESC)^5^ recommend that the most appropriate pharmacological and interventional management should be determined after comprehensive risk assessment.

Many risk models of in‐hospital or short‐term mortality have been developed among patients with acute coronary syndrome.^6^, ^7^, ^8^, ^9^, ^10^, ^11^ Among them, the Global Registry in Acute Coronary Events (GRACE) score is the most commonly used to predict short‐term death.^12^ However, the GRACE score was developed at a time when patient characteristics and management differed significantly from current practice, and few participants were from Asia. Therefore, and attending to the lack of research and the need to update the existing models in this specific population, the purpose of our study was to develop a multivariable COX regression model to better predict short‐term mortality risk among patients with AMI in rural China. We hope that our results will be very valuable to assist clinicians in early identification of high‐risk patients, to reduce in turn the mortality associated with acute coronary syndrome in this underserved population.

## 2 MATERIALS AND METHODS

### 2.1 Study subjects

This observational, retrospective study was conducted at Linyi People's Hospital from January 2013 to December 2018. It included patients with AMI and collected data on patient's demographics, clinical presentations, medical history, risk factors, treatment, and clinical outcomes. The study protocol was approved by the Linyi People’ Hospital Ethics Committee. The study included patients with ST‐segment elevation myocardial infarction (STEMI) as well as those presenting with non‐ST‐elevation myocardial infarction (NSTEMI), in accordance with the third universal definition of myocardial infarction.^13^

Exclusion criteria: patients with active inflammation, liver failure or renal failure on admission; patients with critical data missing; patients lost to follow‐up.

A total of 7533 patients were enrolled in this study: Patients enrolled in 2013–2017 were assigned into a derivation cohort (*n* = 6303) to establish the multivariable COX regression model, and patients enrolled in 2018 were assigned into a validation cohort (*n* = 1230) to validate the risk score. The patients in the derivation cohort and the validation cohort were proved to be homogeneous and comparable (shown in Table S1).

**FIGURE 1:**
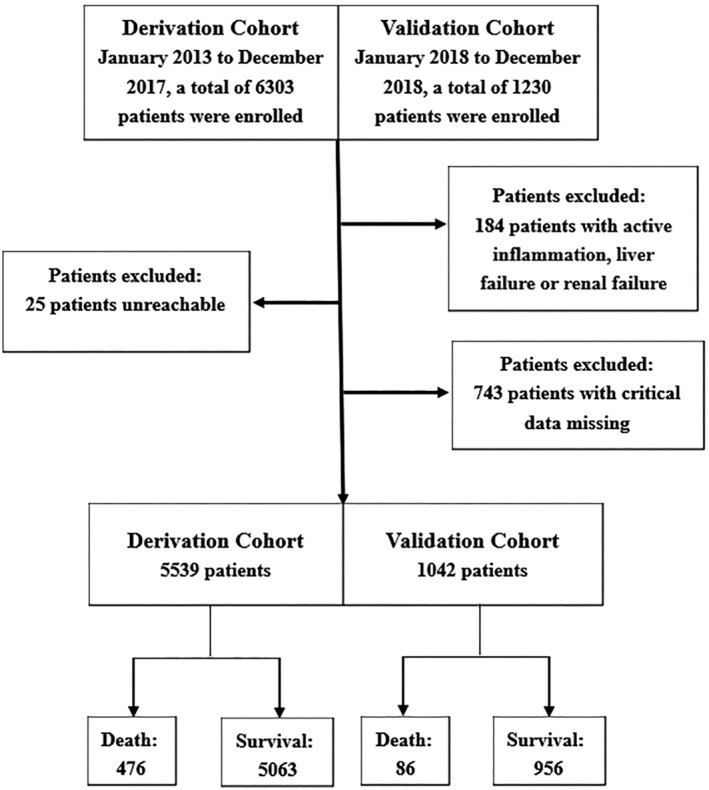
Study flow chart. From January 2013 to December 2018, a total of 7533 patients were enrolled in this study. After excluding 184 patients with active inflammation, liver failure or renal failure; 743 patients due to critical data missing; 25 patients lost to follow, we finally included 6581 AMI patients. A total of 562 patients died in this study sample

As per the exclusion criteria, 184 patients with active inflammation, liver failure or renal failure, 743 patients for which critical data were missing, and 25 patients lost to follow‐up were excluded from analysis. We finally included 6581 AMI patients, of which 562 died over the course of the study. As shown in Figure 1.

### 2.2 Outcome assessment and clinical definitions

The primary endpoint was all‐cause short‐term death, defined as cardiac or non‐cardiac death from admission to 6‐month follow‐up. None of the deaths recorded over the course of this study were caused by accidental injuries such as trauma or car accidents.

Medical history and vital signs were determined at the time of first hospital presentation. Standard definitions of clinical history and physical examination parameters were applied as described in the ACC/AHA Task Force on clinical Data Standards^14^, ^15^, ^16^, ^17^and the National Cardiovascular Data Registry/Acute Coronary Treatment and Intervention Outcomes Network‐Get with the Guidelines' (ACTION–GWTG) data element dictionary.^18^, ^19^

Electrocardiogram (ECG)s and echocardiograms were interpreted locally. Transthoracic Doppler echocardiography (Philips IE33 xMatrix, Amsterdam, The Netherlands) was performed at Linyi People’ Hospital within 48 h after admission. Pulmonary artery systolic pressure (PASP) was calculated by the modified Bernoulli equation. Right atrial pressure was estimated according to the size and respiratory variation of the inferior vena cava diameter in the subcostal view.^20^, ^21^ Left ventricular ejection fraction (LVEF) was calculated using the method of de Simone^22^ complemented by the visual assessment of LV systolic function.

All the variables assessed in this study were collected from electronic medical records. After hospital discharge, clinical end point information was acquired by telephone follow‐up.

### 2.3 Statistical analysis

Statistical analysis was performed using IBM SPSS Statistics for Windows version 21 (IBM Corp., Armonk, New York, United States of America). The distribution pattern of the variables was analyzed using the Kolmogorov–Smirnov test. Continuous variables are presented as mean ± *SD* or median (25th and 75th percentiles). Parametric and non‐parametric continuous variables were compared using Student's *t* test and Mann–Whitney *U* test, respectively. Categorical variables were compared using Pearson χ^2^ test, and the results expressed as percentages. All tests were two‐sided; p ≤ .05 or a 95% confidence interval (CI) that did not include 1.0 indicated significance.

Univariate Cox regression was performed to examine the association between individual baseline variables and short‐term mortality, described as hazard ratio (HR) and 95% confidence interval (CI). For prediction of short‐term mortality risk, the optimized risk model was created by fitting a multivariable COX model to clinical, laboratory analysis, medical history, and treatment variables. All variables that achieved significance (p ≤ .05) on univariable selection were selected to fit the multivariable COX regression model. Then, a stepwise forward selection process was used to identify independent predictors of short‐term death. After selection, the variables with p ≤ .05 were retained in the final model.

The optimized risk score was developed by assigning an integer number to each variable according to their estimated coefficients. The variable with the smallest estimated coefficient was attributed one point and was considered as the reference variable. The scores of other variables were determined by dividing their estimated coefficients by the coefficient of the reference variable (Supplementary Methods).^23^ Receiver operating characteristic (ROC) curves were constructed to assess the discrimination of the model. Z test was applied to compare the differences between the two scoring methods.

After the optimized risk score was established, we compared it with the GRACE score,^12^ which was evaluated before discharge to predict the risk of 6‐month mortality.

## 3 RESULTS

### 3.1 Baseline characteristics

Baseline characteristics for all the patients are shown in Table 1.Compared with survivors, patients who died were older, more often female, and had extensive anterior myocardial infarction. They showed also a greater incidence of polymorphic ventricular arrhythmia, and had a higher Killip class than survivors. Among the dead patients, 95 (16.9%) received Percutaneous coronary intervention (PCI) treatment. Among survivors, 2774 (46.1%) were treated with PCI. The short‐term death group had higher heart rate and lower systolic and diastolic blood pressure than survivors. Patients who died also had more comorbidities, that is, higher prevalence of cerebral infarction, atrial/ventricular arrhythmia, chronic kidney disease, diabetes mellitus, and previous heart failure.

**TABLE 1:** Baseline characteristics between short‐term deaths vs. survivors

Variables	Short‐term deaths (*n* = 562)	Short‐term survivors (*n* = 6019)	Overall population (*n* = 6581)	p value
Baseline demographic characteristics				
Age (years)	72 (64,79)	63 (53,77)	64 (54,77)	<.001
Male (%)	311 (55.4)	4201 (69.8)	4512 (68.6)	<.001
STEMI (%)	241 (42.8)	2751 (45.7)	2992 (45.4)	.091
Extensive anterior myocardial infarction (%)	175 (31.1)	1126 (18.7)	1301 (19.8)	<.001
Anterior myocardial infarction (%)	111 (19.7)	1336 (22.2)	1447 (21.9)	0.449
Inferior myocardial infarction (%)	220 (39.2)	2486 (41.3)	2706 (41.1)	0.361
Ventricular arrhythmia (%)				<.001
Polymorphic	164 (29.2)	664 (11)	828 (12.6)	
Monomorphic	121 (21.5)	1726 (28.7)	1847 (28.1)	
Admission characteristics				
Heart rate (beat/minute)	82 (79102)	76 (64,86)	74 (65,87)	.002
SBP (mmHg)	118 (99135)	129 (112145)	129 (112144)	.007
DBP (mmHg)	74 (64,83)	78 (68,87)	77 (68,86)	.004
Killip classification (%)				<.001
I	144 (25.6)	3967 (65.9)	4111 (62.5)	
II	152 (27)	1204 (20)	1356 (20.6)	
III	117 (20.9)	590 (9.8)	707 (10.7)	
IV	149 (26.5)	258 (4.3)	407 (6.2)	
Past medical history (%)				
Hypertension	275 (48.9)	2739 (45.5)	3014 (45.8)	0.12
Diabetes mellitus	156 (27.7)	1348 (22.4)	1504 (22.9)	.002
Arrhythmia				<.001
atrial arrhythmia	63 (11.2)	421 (7)	484 (7.4)	
ventricular arrhythmia	11 (2)	55 (0.9)	66 (1.0)	
Heart failure				<.001
HFrEF	10 (1.8)	57 (0.9)	67 (1.0)	
HFmrEF	19 (3.4)	104 (1.7)	123 (1.9)	
HFpEF	6 (1.1)	92 (1.5)	98 (1.5)	
Previous myocardial infarction	42 (7.4)	355 (5.9)	397 (6.0)	0.23
Smoking status				0.243
Ex‐smoker	144 (25.6)	1408 (23.4)	1552 (23.6)	
Current smoker	119 (21.2)	1252 (20.8)	1371 (20.8)	
COPD history	36 (6.4)	271 (4.5)	307 (4.7)	.096
Cerebral infarction	80 (14.2)	632 (10.5)	712 (10.8)	.021
Chronic kidney disease	19 (3.4)	78 (1.3)	97 (14.7)	.001
Laboratory tests results				
WBC (109/L)	10.8 (8.7,14.3)	9.5 (7.4,11.9)	9.7 (7.5,12.0)	<.001
Admission blood glucose (mmol/L)	8.7 (5.7,10.5)	7.1 (5.0,7.9)	7.2 (5.1,7.9)	<.001
Creatinine (umol/L)	95.2 (71.1126.7)	68.1 (58.4,82.0)	69.5 (58.8,82.2)	<.001
Troponin T (μg/L)	2.0 (0.2,4.5)	1.8 (0.1,2.2)	1.9 (0.1,3.8)	0.115
Cardiac color Doppler				
LVEDD (mm)	49 (47,55)	49 (45,52)	49 (46,53)	.087
LAD (mm)	35 (33,38)	34 (31,36)	35 (32,37)	<.001
PASP (mmHg)	33 (27,38)	29 (25,34)	29 (25,35)	<.001
LVEF value (%)	49 (45,51)	53 (46,58)	52 (46,56)	<.001
Invasive treatment and medication in hospital				
PCI treatment (%)	95 (16.9)	2774 (46.1)	2869 (43.6)	<.001
Aspirin (%)	523 (93.1)	5772 (95.9)	6295 (95.7)	.004
Clopidogrel (%)	451 (80.2)	4966 (82.5)	5417 (82.3)	0.168
Double antiplatelet therapy (%)	443 (78.9)	4972 (82.6)	5415 (82.3)	.054
Statins (%)	497 (88.4)	5441 (90.4)	5938 (90.2)	.067
Ticagrelor (%)	60 (10.7)	752 (12.5)	812 (12.3)	0.239
β‐receptor Antagonists (%)	327 (58.3)	4340 (72.1)	4667 (70.9)	<.001
ACEI/ARB (%)	243 (43.3)	3437 (57.1)	3680 (55.9)	<.001
Diuretics (%)	256 (45.6)	2010 (33.4)	2266 (34.4)	<.001
Antibiotics (%)	141 (25)	999 (16.6)	1140 (17.3)	<.001
Vasoactive drugs (%)	78 (13.8)	337 (5.6)	415 (6.3)	<.001

*Note*: Vasoactive drugs include: epinephrine, norepinephrine, dopamine, meta‐hydroxylamine.

Abbreviations: ACEI/ARB, angiotensin‐converting enzyme inhibitors/angiotensin receptor blockers; COPD: chronic obstructive pulmonary disease; DBP, diastolic blood pressure; HFrEF, Heart failure with reduced ejection fraction; HFmrEF, Heart failure with mid‐range ejection fraction; HFpEF, Heart failure with preserved ejection fraction; HR, heart rate; LVEDD, left ventricular end‐diastolic diameter; LAD, left atrium diameter; LVEF, left ventricular ejection fraction; PCI, percutaneous coronary; intervention; SBP, systolic blood pressure; STEMI, ST‐segment elevation myocardial infarction; PASP, pulmonary artery systolic pressure; WBC, white blood cell.

On laboratory analysis, patients who died had higher white blood cell (WBC) count, blood glucose, and serum creatinine. On cardiac color doppler ultrasound, non survivors had higher left atrium anteroposterior diameter (LAD), higher PASP, and lower LVEF values. The utility of antibacterial drugs and vasoactive drugs was higher, while the utility of aspirin, low‐molecular‐weight heparin, lipid‐lowering drugs, nitrate drugs, β‐receptor antagonists, and Angiotensin‐converting enzyme inhibitor / Angiotensin Receptor Blocker (ACEI/ARB) drugs was lower for dead patients.

### 3.2 Independent predictors of short‐term death

Univariate Cox regression was performed to examine the association between individual baseline variables and short‐term mortality (Table S2). All variables that achieved significance (p ≤ .05) were selected to fit the multivariable COX regression model.

A total of 25 variables with p ≤ .05 were selected to fit the multivariable COX regression model: age; gender; presence of extensive anterior myocardial infarction; presence of ventricular arrhythmia; whether treated with PCI; heart rate; systolic blood pressure; diastolic blood pressure; Killip classification at admission; previous cerebral infarction; arrhythmia; diabetes mellitus; heart failure; chronic kidney disease; white blood cell count; blood glucose; creatinine level; PASP; LVEF; aspirin use; antibiotics use; use of vasoactive drugs; use of β receptor antagonists; use of ACEI/ARB drugs; and use of diuretics. After stepwise selection, a total of six variables with a p ≤ .05 were identified as independent predictors of short‐term death: age, PCI treatment, Killip IV class, blood glucose, serum creatinine, and PASP (Table S3).

### 3.3 Optimized scoring method

**FIGURE 2:**
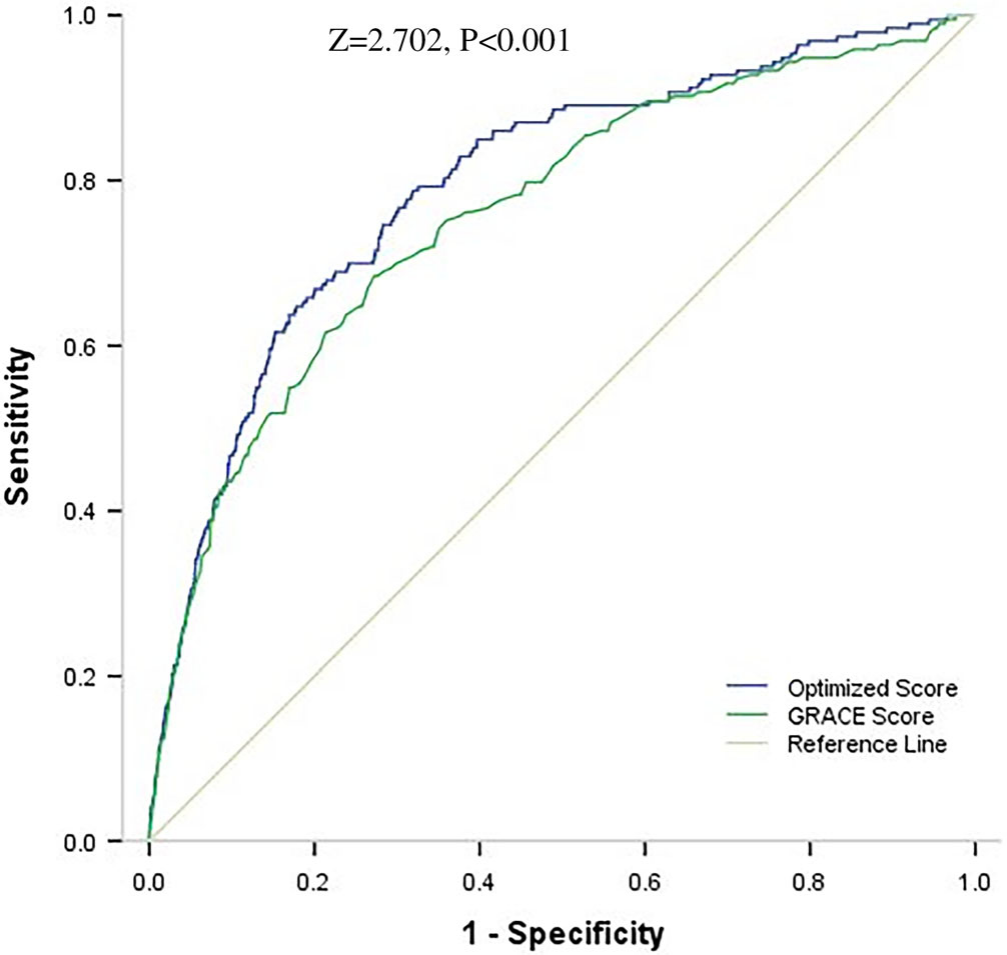
ROC curves of Optimized risk score and GRACE risk score within derivation cohort. Area under curve value was 0.82 (95% CI: 0.78‐0.85) for Optimized risk score and 0.76 (95% CI: 0.73‐0.79) for GRACE risk score

**FIGURE 3:**
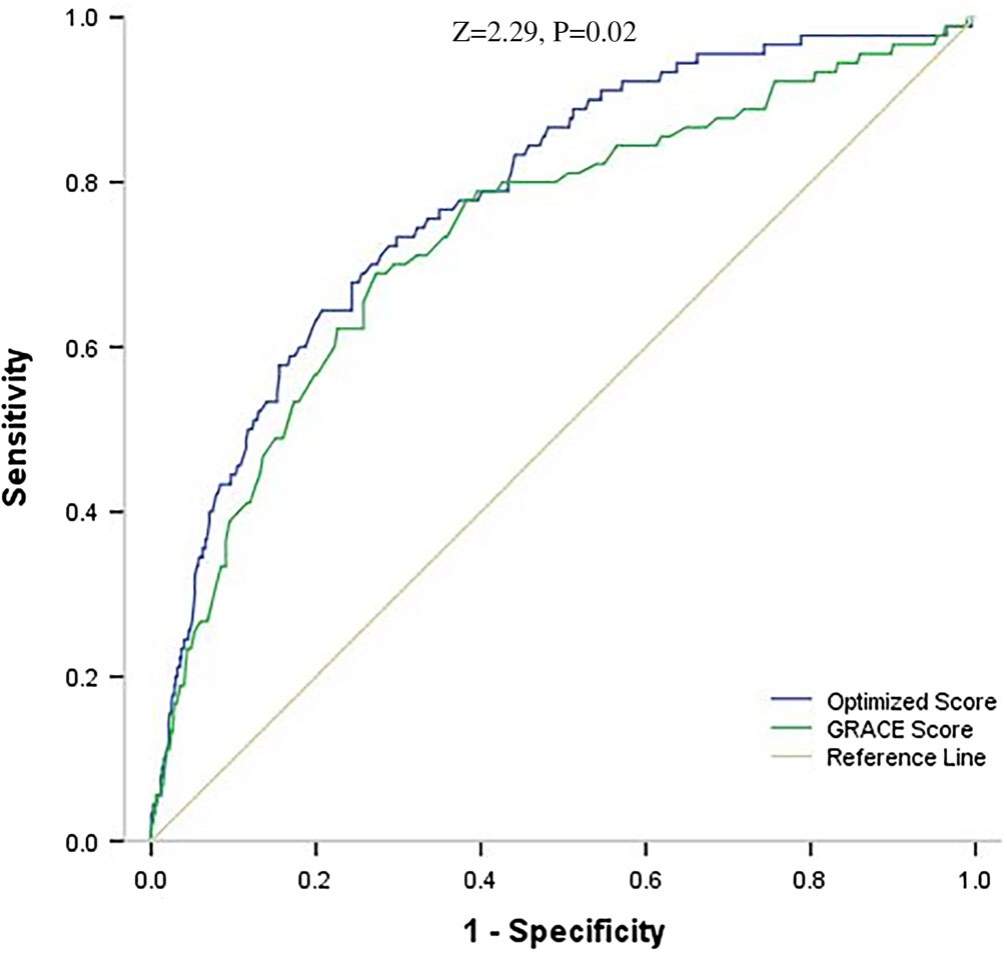
ROC curves of Optimized risk score and GRACE risk score within validation cohort. Area under curve value was 0.81 (95% CI: 0.77–0.84) for Optimized risk score and 0.75 (95% CI: 0.71–0.78) for GRACE risk score

We developed a simplified risk score by attributing an integer number to each variable according to their estimated coefficients (Table 2). The optimized score thus obtained ranges from 0 to 254, and the corresponding short‐term death risk ranges from 0.3% to 97.7%. Within the derivation cohort (*n* = 5539), AUC values for the optimized score and the GRACE score were 0.82 (95% CI: 0.78–0.85) and 0.76 (95% CI: 0.73–0.79), respectively. The AUC of the optimized score was significantly better than that of the GRACE score (Z value = 3.7, p < .001) (Figure 2). Within the validation cohort, (*n* = 1042) AUC values for the optimized and the GRACE scores were 0.81 (95% CI: 0.77–0.84) and 0.75 (95% CI: 0.71–0.78), respectively. In this cohort, the AUC of the optimized score was also significantly better than that of the GRACE score (Z value = 2.29, p = .02) (Figure 3).

**TABLE 2:** Scores attributed to each variable

Variable		Point	Variable		Point
Age (years)	<40	0	4. Blood glucose (mmol/L)	<6.1	0
	[40–50)	20		[6.1–8.1)	3
	[50–60)	36		[8.1–10.1)	5
	[60–70)	52		[10.1–14.1)	9
	[70–80)	68		[14.1–20.1)	15
	[80–89)	84		>20.1	23
	>90	100	5. Creatinine (umol/L)	<60	0
2. Killip classification	I	0		[60 < 80)	2
	II	19		[80–120)	4
	III	39		[120–160)	6
	IV	59		[160–200)	10
3. PASP(mmHg)	<25	0		[200–360)	15
	[25–35)	10		>360	21
	[35–45)	18	6.PCI treatment	yes	0
	>45	25		no	26

Abbreviations: PASP, pulmonary artery systolic pressure; PCI, percutaneous coronary intervention.

We then divided all participants within the derivation cohort into quartiles based on the corresponding risk scores. Each quartile contained approximately one fourth of the population (Table S4). Event rate increased significantly across quartiles: 1.44% in Quartile I (score range: 0–83), 3.42% in Quartile II (score range: 84–111), 7.17% in Quartile III (score range: 112–152), and 22.34% in Quartile IV (score range: ≥153).

A similar pattern was observed within the validation cohort, as the event rate also increased significantly across quartiles: 1.6% in Quartile I, 3.11% in Quartile II, 7.29% in Quartile III, and 20.07% in Quartile IV. Therefore, we defined Quartile I, II, III, and IV as low, intermediate, high, and extremely high risk groups respectively.

## 4 DISCUSSION

In this large‐scale contemporary retrospective study of patients with AMI from rural China, we identified six independent predictors of short‐term death and used these variables to develop and validate a risk prediction tool for short‐term death among AMI patients. The optimized score, which was designed for rapid risk assessment after presentation, had high discrimination ability in both the derivation and validation cohorts. A significant gradient of increasing short‐term mortality risk was identified as the optimized score increased.

Many risk prediction tools have been developed to assess short‐ or long‐term death risk for acute coronary syndrome (ACS) patients. Among these, the GRACE risk score, proposed in 2003, remains the most popular and validated model. Therefore, and since our novel scoring method shares a similar study object and primary endpoint event as the GRACE risk model, we used the latter to contrast and validate the predictive power of the optimized risk score model herein described.

From 1999 to 2008, the profile of AMI patients changed over time, with a slight increase in NSTEMI and a decrease in STEMI cases.^24^ In turn, the incidence and short‐ and long‐term mortality rates of AMI were impacted by an updated diagnostic criteria introduced in 2014.^25^, ^26^, ^27^ In parallel, mortality rates for both STEMI and NSTEMI patients have declined significantly due to improved medication management and optimization of invasive treatments.^28^ However, the above population trends, as well as the GRACE risk score, were largely based on clinical data from Western populations and included only few Asian cases. Therefore, the prognostic significance of current risk score models in Asian patients may be diminished by both actual and potential differences in lifestyle habits and disease etiology.

Several variables in the present model, including age, Killip class, serum creatinine, and PCI, are consistent with previous prediction tools. Compared with previous studies, the present model incorporated two new variables: blood glucose and PASP. Abnormal glucose metabolism is a common feature in patients with CAD. Previous studies have shown that blood glucose levels are commonly elevated in early AMI and represent an independent risk factor for increased in‐hospital or short‐term mortality.^18^, ^19^ However, the impact of blood glucose in the context of MI is complex, involving both protective and deleterious effects on the myocardium.^29^ This may explain why blood glucose was not included in the GRACE or CAMI risk scores.^10^, ^30^

Pulmonary hypertension is frequently observed following AMI,^31^ and elevated PASP after AMI is often associated with poor prognosis.^32^, ^33^ Indeed, PASP was proved to be a strong independent predictor of short‐term death in a previous study.^34^ These findings imply that special attention must be paid to elevated PASP during comprehensive evaluation of cardiac function in AMI patients.

In our multivariable COX model, the HRs of blood glucose and PASP were 1.046 and 1.023 respectively, indicating that neither of them can be used individually to determine the risk of adverse events. Nonetheless, the diagnostic performance of the risk score is improved by addition of blood glucose and PASP into the risk model.

LVEF in our study merits emphasis. The median value was 49% even in the death group, and clinicians tended to ignore the patients' heart function because of this indicator. PASP was always reported in articles about HF. It was reported that approximately one‐half of patients with HF have a preserved ejection fraction.^35^ Recent studies have shown that a significant number of patients who display right ventricular dysfunction (including increased PASP) have high morbidity and mortality.^36^, ^37^ So, the heart function of patients with elevated PASP in this study could be considered impaired after AMI. But it had not attracted enough attention as there was no obvious clinical heart failure manifestation.

In this study, PCI was the only improving factor, and the PCI rate was lower in the non‐survivor group. The 2011 American College of Cardiology Foundation/American Heart Association/the Society for Cardiovascular Angiography and Interventions (ACCF/AHA/SCAI) Guideline for Percutaneous Coronary Intervention^38^ was used to perform PCI in this study. There were 47.4% of patients with Killip class III to IV in the non‐survivor group; the difficulties of successful implementation of PCI in these critically ill patients are well known. On the other hand, our hospital is located in a remote rural area and has limited medical resources and technology; therefore, the PCI rate is lower than the country's average. The population sample in this study draws as well from rural areas. For these AMI patients, pre‐hospital delays were typically longer, most could not afford high medical expenses, and PCI rates were lower. Many patients would be discharged from the hospital and abandon treatment after realizing that the disease is very severe and carries a poor prognosis. Accordingly, patients that presented short‐term mortality had a quite low PCI rate.

Notwithstanding, standardized protocols were followed in all the patients that eventually received PCI or medication treatment. Many patients in the non‐survivor group had cardiogenic shock, pulmonary edema, and lung infections, so in this group the use of antibacterial drugs and vasoactive drugs was higher. Many patients in this group were also older than 80 years, or had complications such as stress ulcers, abnormal liver and kidney function, and ventricular tachycardia and hypotension. Thus, for non‐survivors the utility of PCI, aspirin, low‐molecular‐weight heparin, lipid‐lowering drugs, nitrate drugs, β‐receptor antagonists, and ACEI/ARB drugs was lower.

In recent years, a rise in the morbidity and mortality of the rural population in China has been apparent. We hope that our study can provide a reference for larger studies in the future and serve as a model to identify causative factors driving increased disease incidence and mortality in rural China. The risk score system presented here is simple, easy to implement, and includes objective indicators. We thus believe that adopting our scoring method will significantly improve prediction of short‐term risk of death in AMI patients.

Our study has some limitations. First, this is a retrospective study and all the patients included were assessed at a single research center in China. Second, although the discrimination ability of the optimized risk score was confirmed in a separate cohort of patients, its predictive accuracy for short‐term death should be further validated using a larger population sample. In addition, whether this risk score can be extrapolated to different ethnicities requires further validation.

In summary, we developed and validated a risk score model to predict short‐term death risk in patients with AMI. Our optimized risk score proved to be superior, in our large AMI cohort, to the widely used GRACE risk model. We expect that this optimized risk model will provide clinicians with a simple and useful tool to accurately assess short‐term death risk and to select appropriate treatment and level of care for AMI patients.

## CONFLICT OF INTEREST

The authors declare no conflicts of interest regarding this manuscript.

## Supporting information


**Table S1** Baseline characteristics between derivation cohort vs. validation cohort
**Table S2** Univariate analysis of the association between baseline characteristics and short‐term mortality
**Table S3** Independent predictors of short‐term death among AMI patients
**Table S4** Event rate Across Different Risk Group

## Data Availability

Supplementary information is not publicly available but is available from corresponding author on reasonable request.
