# Supplementary material for: Development of an optimized risk score to predict short‐term death among acute myocardial infarction patients in rural China
Source: Clin Cardiol. 2021 Mar 25;44(5):699–707. doi: 10.1002/clc.23598 (PMC8119840; doi:10.1002/clc.23598)
Supplement: Supplementary file 1 — Table S1 Baseline characteristics between derivation cohort vs. validation cohort Table S2 Univariate analysis of the association between baseline characteristics and short‐term mortality Table S3 Independent predictors of short‐term death among AMI patients Table S4 Event rate Across Different Risk Group [file CLC-44-699-s001.docx]

|  | Supplementary Table1 Baseline characteristics between derivation cohort vs. validation cohort | | | | | |
| --- | --- | --- | --- | --- | --- | --- |
| Variables | | | derivation cohort  （n=5539） | validation cohort（n=1042） | P value |  |
| Baseline demographic characteristics | | | |  |  |  |
| Age (years) | | | 64(53,77) | 64(54,78) | 0.968 |  |
| Male (%) | | | 3833(69.2) | 718(68.9) | 0.975 |  |
| STEMI (%) | | | 2576(46.5) | 476(45.7) | 0.942 |  |
| Extensive anterior myocardial infarction (%) | | | 504(9.1) | 94(9) | 0.983 |  |
| Anterior myocardial infarction (%) | | | 565(10.2) | 107(10.3) | 0.996 |  |
| Inferior myocardial infarction (%) | | | 1063(19.2) | 196(18.8) | 0.784 |  |
| Ventricular arrhythmia (%) | | | 864(15.6) | 158(15.2) | <0.001 |  |
| Admission characteristics | | | |  |  |  |
| Heart rate (beat/minute) | | | 74(64,87) | 75(65,88) | 0.852 |  |
| SBP (mmHg) | | | 129(112,144) | 128(111,142) | 0.892 |  |
| DBP (mmHg) | | | 77(68,87) | 76(65,86) | 0.934 |  |
| Killip classification (%) | | |  |  | 0.841 |  |
| Ⅰ | | | 3456(62.4) | 671(64.4) |  |  |
| Ⅱ | | | 1141(20.6) | 189(18.1) |  |  |
| Ⅲ | | | 598(10.8) | 121(11.6) |  |  |
| Ⅳ | | | 344(6.2) | 61(5.9) |  |  |
| Past medical history (%) | | |  |  |  |  |
| Hypertension | | | 2531(45.7) | 481(46.2) | 0.691 |  |
| Diabetes mellitus | | | 1213(21.9) | 242(23.2) | 0.832 |  |
| Arrhythmia | | | 438(7.9) | 84(8.1) | 0.787 |  |
| Heart failure | | | 249(4.5) | 46(4.4) | 0.954 |  |
| Previous myocardial infarction | | | 321(5.8) | 64(6.1) | 0.919 |  |
| Smoking status | | | 2454(44.3) | 445(42.7) | 0.694 |  |
| COPD history | | | 316(5.7) | 61(5.9) | 0.096 |  |
| Cerebral infarction | | | 609(11) | 111(10.7) | 0.738 |  |
| Chronic kidney disease | | | 100(1.8) | 20(1.9) | 0.889 |  |
| Laboratory tests results | | |  |  |  |  |
| WBC (109/L) | | | 9.7(7.4,12.0) | 9.9(7.5,12.2) | 0.832 |  |
| Admission blood glucose(mmol/L) | | | 7.2(5.1,7.9) | 7.3(5.3,8.2) | 0.915 |  |
| Creatinine (umol/L) | | | 68.8(58.5,82.1) | 69.4(57.4,82.5) | 0.857 |  |
| Troponin T (μg/L) | | | 1.9(0.1,3.7) | 2.0(0.1,3.9) | 0.879 |  |
| Cardiac color Doppler | | |  |  |  |  |
| LVEDD (mm) | | | 49(46,52) | 50(46,53) | 0.944 |  |
| LAD (mm) | | | 35(32,37) | 35(33,38) | 0.833 |  |
| PASP (mmHg) | | | 29(25,34) | 30(26,35) | 0.798 |  |
| LVEF value (%) | | | 51(46,55) | 50(45,55) | 0.921 |  |
| Invasive and pharmacological treatment in hospital | | | | |  |  |
| PCI treatment (%) | | | 2387(43.1) | 475(45.6) | 0.743 |  |
| Aspirin (%) | | | 5295(95.6) | 998(95.8) | 0.895 |  |
| Clopidogrel (%) | | | 4592(82.9) | 862(82.7) | 0.861 |  |
| Statins(%) | | | 5306(95.8) | 999(95.9) | 0.874 |  |
| Ticagrelor (%) | | | 654(11.8) | 128(12.3) | 0.239 |  |
| β-receptor Antagonists (%) | | | 3927(70.9) | 763(73.2) | 0.941 |  |
| ACEI/ARB (%) | | | 3118(56.3) | 602(57.8) | 0.835 |  |
| Diuretics(%) | | | 1916(34.6) | 367(35.2) | 0.913 |  |
| Antibiotics (%) | | | 986(17.8) | 176(16.9) | 0.799 |  |
| Vasoactive drugs (%) | | | 354(6.4) | 60(5.8) | 0.851 | |

**Abbreviations:** STEMI, ST-segment elevation myocardial infarction; PCI, percutaneous coronary

intervention; HR, heart rate; SBP, systolic blood pressure; DBP, diastolic blood pressure; COPD: chronic obstructive pulmonary disease; HFrEF, Heart failure with reduced ejection fraction; HFmrEF , Heart failure with mid-range ejection fraction; HFpEF, Heart failure with preserved ejection fraction; WBC: white blood cell; LVEDD, left ventricular end-diastolic diameter; LAD, left atrium diameter; PASP, pulmonary artery systolic pressure; LVEF, left ventricular ejection fraction; ACEI/ARB, angiotensin-converting enzyme inhibitors/angiotensin receptor blockers.

**Note:** Vasoactive drugs include: epinephrine, norepinephrine, dopamine, meta-hydroxylamine.

| Supplementary Tables 2 Univariate analysis of the association between baseline characteristics and short-term mortality | | |
| --- | --- | --- |
| Variable | HR(95%CI) | P value |
| Age, every additional year | 1.057（1.049-1.065） | <0.001 |
| Male | 0.55（0.461-0.658） | <0.001 |
| Extensive anterior wall myocardial infarction | 1.655（1.224-2.237） | 0.001 |
| Ventricular arrhythmia | 2.058（1.39-3.046） | <0.001 |
| PCI treatment | 0.232（0.182-0.296） | <0.001 |
| Heart rate，every additional beat | 1.009（1.004-1.014） | <0.001 |
| SBP，every additional 1 mmHg | 0.995（0.991-0.999） | 0.015 |
| DBP，every additional 1 mmHg | 0.991（0.984-0.998） | 0.008 |
| Killip classification |  |  |
| Ⅱ vs. Ⅰ | 3.3（2.58-4.22） | <0.001 |
| Ⅲ vs. Ⅰ | 5.478（4.235-7.087） | <0.001 |
| Ⅳ vs. Ⅰ | 12.863（10.049-16.464） | <0.001 |
| Cerebral infarction | 1.474(1.153-1.885) | 0.002 |
| Previous arrhythmia | 1.815（1.395-2.36） | <0.001 |
| Diabetes mellitus | 1.363（1.118-1.661） | 0.002 |
| Heart failure | 1.494(1.044-2.138) | 0.028 |
| Chronic kidney disease | 2.796（1.746-4.476） | <0.001 |
| WBC, every additional 1*109/L | 1.055（1.034-1.076） | <0.001 |
| Blood glucose，every additional 1mmol/L | 1.07（1.058-1.083） | <0.001 |
| Creatinine level，every additional 1umol/L | 1.005（1.004-1.006） | <0.001 |
| PASP，every additional 1mmHg | 1.027（1.02-1.034） | <0.001 |
| LVEF，every additional1% | 0.967（0.957-0.978） | <0.001 |
| Aspirin | 0.573（0.406-0.808） | 0.001 |
| Vasoactive drugs | 2.619（2.034-3.373） | <0.001 |
| Antibiotics | 1.72（1.408-2.101） | <0.001 |
| β receptor antagonists, | 0.542（0.453-0.648） | <0.001 |
| ACEI/ARB | 0.634（0.531-0.758） | <0.001 |
| Diuretics | 1.504(1.258-1.797) | <0.001 |

**Abbreviations:** HR, hazard ratio; PCI, percutaneous coronary intervention; SBP, systolic blood pressure; DBP, diastolic blood pressure; WBC: white blood cell; PASP, pulmonary artery systolic pressure; LVEF, left ventricular ejection fraction; ACEI/ARB, angiotensin-converting enzyme inhibitors/angiotensin receptor blockers.

| Supplementary Table 3 Independent predictors of short-term death among AMI patients | | |
| --- | --- | --- |
| Variable | Multivariate analysis | |
|  | HR(95%CI) | P value |
| Age (years) | 1.041（1.011-1.072） | 0.007 |
| PCI treatment | 0.349（0.144-0.845） | 0.02 |
| Killip Ⅳ vs. Ⅰ | 3.28（1.304-8.255） | 0.012 |
| Blood glucose | 1.046（1.006-1.087） | <0.001 |
| Creatinine | 1.005（1.001-1.008） | 0.005 |
| PASP | 1.023（1.002-1.045） | 0.041 |

**Abbreviations:** HR, hazard ratio; PCI, percutaneous coronary intervention; PASP, pulmonary artery systolic pressure.

|  | Low Risk Group  (Quartile Ⅰ) | Intermediate Risk Group  (Quartile Ⅱ) | | High Risk Group  (Quartile Ⅲ) | Extremely High Risk Group  (Quartile Ⅲ) | P value |
| --- | --- | --- | --- | --- | --- | --- |
| Score range | 0-83 | 84-110 | | 111-152 | ≥153 |  |
| Short-term mortality rate | | |  |  |  |  |
| Derivation Cohort | 1.4% (20/1385) | 3.4% (47/1376) | | 7.2% (100/1395) | 22.3% (309/1383) | <0.001 |
| Validation Cohort | 1.6%（4/250） | 3.1%（8/257） | | 7.3%（19/261） | 20.1%（55/274） | <0.001 |

Supplementary Table 4 Event rate Across Different Risk Group
